# Maternal Exposure to Bisphenol A Combined with High-Fat Diet-Induced Programmed Hypertension in Adult Male Rat Offspring: Effects of Resveratrol

**DOI:** 10.3390/ijms20184382

**Published:** 2019-09-06

**Authors:** Chien-Ning Hsu, Yu-Ju Lin, You-Lin Tain

**Affiliations:** 1 Department of Pharmacy, Kaohsiung Chang Gung Memorial Hospital, Kaohsiung 833, Taiwan; 2 Department of Obstetrics and Gynecology, Kaohsiung Chang Gung Memorial Hospital and Chang Gung University College of Medicine, Kaohsiung 833, Taiwan; 3 Departments of Pediatrics, Kaohsiung Chang Gung Memorial Hospital and Chang Gung University College of Medicine, Kaohsiung 833, Taiwan

**Keywords:** asymmetric dimethylarginine, aryl hydrocarbon receptor, bisphenol A, developmental origins of health and disease (DOHaD), high-fat diet, hypertension, nitric oxide, oxidative stress

## Abstract

Maternal exposure to endocrine disrupting chemicals (EDCs) and a high-fat intake may induce the developmental programming of hypertension in adult offspring. Bisphenol A (BPA) is one of the most commonly environmental EDCs. As the nitric oxide (NO) and aryl hydrocarbon receptor (AHR) signaling pathways both contribute to the pathogenesis of hypertension, we evaluated whether resveratrol, an antioxidant and an AHR antagonist, can prevent hypertension programmed by a maternal BPA and HF diet. Sixteen-week-old male rat offspring were assigned to six groups (n = 8 per group): Control, HF (D12331, Research Diets), BPA (50 μg/kg/day), HF + BPA, BPA + R (resveratrol 50mg/L in drinking water throughout pregnancy and lactation), and HF + BPA + R. Maternal BPA exposure exacerbated hypertension programmed by HF consumption in adult male offspring, which was protected by maternal resveratrol therapy. The BPA and HF diet synergistically induced oxidative stress in offspring kidneys, which resveratrol treatment prevented. We observed that HF + BPA-induced programmed hypertension was associated with a decreased NO bioavailability, increased oxidative stress, and an activated AHR signaling pathway. The beneficial effects of resveratrol are relevant to restoring NO bioavailability, reducing oxidative stress, and antagonizing the AHR signaling pathway. Our results cast a new light on resveratrol as a reprogramming strategy to protect against hypertension programmed by combined BPA and HF exposure, but this strategy has yet to be translated into clinical applications.

## 1. Introduction

Maternal exposure to environmental chemicals, such as endocrine disrupting chemicals (EDCs), can increase disease risk later in life. This concept is known as the developmental origins of health and disease (DOHaD) [1]. One of the most commonly environmental EDC exposures is bisphenol A (BPA). Though the U.S. Food and Drug Administration and European Food Safety Authority have banned BPA from infant formula bottles, many plastic food and drinking containers still contain BPA. BPA has been reported to be associated with the development of cardiovascular disease and hypertension in both children and adults [2]. A previous study showed that exposure to BPA during pregnancy is associated with higher blood pressure (BP) of children [3]. However, the impacts of maternal BPA exposure on BP in adult offspring remain unclear.

High-fat (HF) consumption is closely linked to the development of hypertension [4]. Our previous report demonstrated that a maternal plus post-weaning HF diet induced hypertension in adult male offspring [5]. Development is a plastic process that is sensitive to environmental insults including nutrition, stress, drugs, and environmental pollutants. These environmental insults may interact with each other, resulting in an increased susceptibility to similar diseases. Since a second insult or challenge could deteriorate earlier programming effects induced by the first insult, we hypothesized that BPA exposure enhances offspring vulnerability to HF diet-induced programmed hypertension.

BPA acts as an endogenous estrogen by interacting with estrogen receptors [6]. Estrogen acts as a vasodilator by regulating the nitric oxide (NO) system [7]. Additionally, BPA is a ligand for the aryl hydrocarbon receptor (AHR). The AHR is involved in the transcription of distinct genes that are associated with hypertension [8]. Our previous studies suggest that the NO and AHR signaling pathways both contribute to the pathogenesis of hypertension of developmental origins [9,10].

Conversely, several therapeutic strategies by so-called reprogramming have been applied to reverse the programming processes and prevent the development of hypertension [11]. Resveratrol is a naturally occurring phytoalexin found in various plants, especially berry fruits, and it is a popular nutritional supplement. Resveratrol possesses many beneficial effects include anti-inflammatory and antioxidant properties, anti-obesogenic and anti-atherosclerotic effects, anti-carcinogenic activity, the inhibition of platelet aggregation, the improvement of endothelial function, the restoration of NO bioavailability, and the ability to serve as an AHR antagonist [12]. Emerging evidence supports the idea that resveratrol might serve as a reprogramming strategy to prevent a variety of disorders, including hypertension [12]. Additionally, resveratrol has been reported to prevent BSA-induced vascular toxicity [13].

BP is regulated by a complex process that is controlled mainly by the kidney. The developing kidney is vulnerable to suboptimal early-life environments, which may produce renal programming and programmed hypertension [11]. We investigated whether combined maternal BPA and HF exposure induced programmed hypertension in adult male offspring via the activation of AHR signaling and disturbing NO pathways and whether resveratrol can protect against hypertension programmed by combined BPA and HF exposure, with a focus on the kidney.

## 2. Results

### 2.1. Morphometric Values and Blood Pressures

**Figure 1:**
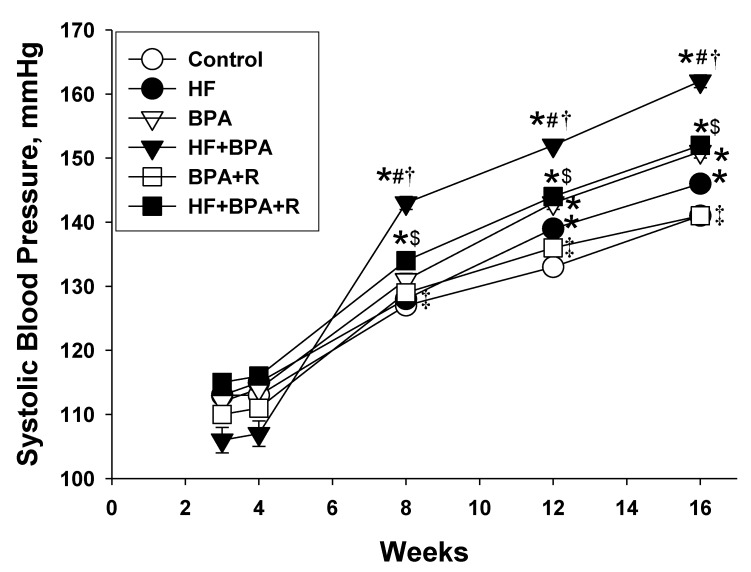
Effects of maternal high-fat diet (HF), bisphenol A (BPA), and resveratrol (R) on systolic blood pressure (SBP) measured in male offspring at 3, 4, 8, 12 and 16 weeks of age. *n* = 7–8/group; * *p* < 0.05 versus control; # *p* < 0.05 versus HF; † *p* < 0.05 versus BPA; ‡ *p* < 0.05 versus HF + BPA; $ *p* < 0.05 versus BPA + R.

Neither a HF diet nor BPA exposure affected the survival of male pups (Table 1). However, one rat pup died soon after birth in the HF + BPA group. The body weight (BW), kidney weight, and kidney weight-to-BW ratio were not different between the six groups. At 16 weeks of age, both HF diet and BPA exposure resulted in the elevation of systolic blood pressure (SBP). BPA exposure further increased 16 mmHg of SBP in the HF + BPA group compared with that in the HF group (Table 1). As shown in Figure 1, SBP was higher in HF + BPA-exposed rats than those in the controls from 8 to 16 weeks of age. Conversely, resveratrol treatment reduced SBP from 10 to 16 weeks and produced a significant reduction of SBP (~10 mmHg) in the HF + BPA + R offspring compared to the HF + BPA group at 16 weeks of age. Similarly, resveratrol treatment caused a reduction of SBP (~10 mmHg) in the BPA + R group vs. the BPA group at 16 weeks of age.

### 2.2. Plasma Levels of NO-Related Elements

Since asymmetric dimethylarginine (ADMA, an endogenous inhibitor of the NO synthase)-related reactive oxygen species (ROS)/NO imbalance plays an important role in programmed hypertension [14], we first examined the elements in the ADMA–NO pathway. l-arginine (the substrate for the NO synthase) and ADMA each competed for NOS and were present in a ratio that maintained NO homeostasis; this ratio defined NO bioavailability [15]. Table 2 shows that combined HF and BPA exposure caused increases of plasma ADMA and symmetric dimethylarginine (SDMA, an isomer of ADMA) levels as well as a decrease in the plasma l-arginine-to-ADMA ratio vs. controls. Resveratrol treatment reduced plasma SDMA levels in the BPA + R group compared to that in the BPA group. Additionally, resveratrol treatment caused higher plasma levels of l-citrulline and l-arginine in the HF + BPA + R group than those in the HF + BPA-treated offspring. These findings indicate that HF + BPA reduced NO bioavailability, which resveratrol treatment prevented. 

### 2.3. Protein Levels in the ADMA–NO Pathway

**Figure 2:**
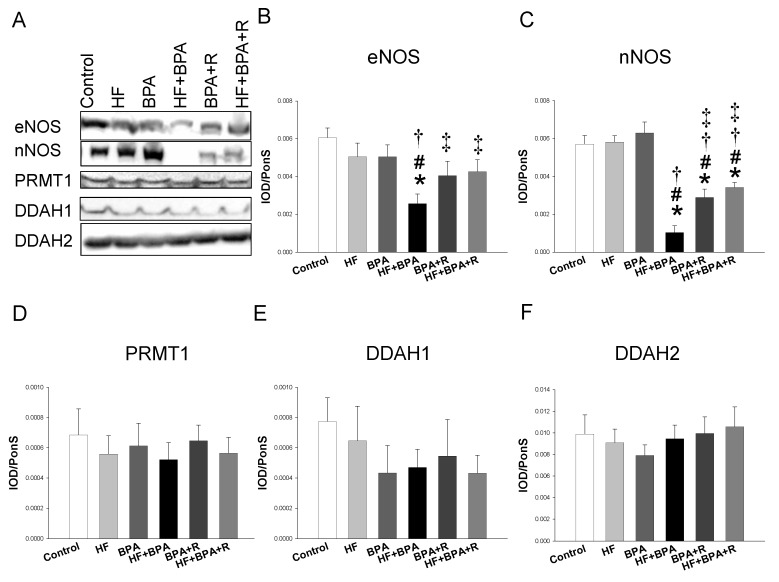
Representative Western blots (**A**) show the endothelial NO synthase (eNOS) (~150 kDa), the neuronal NO synthase (nNOS) (~160 kDa), PRMT1 (~42 kDa), DDAH-1 (~34 kDa), and DDAH-2 (~30 kDa) bands in the offspring kidneys at 16 weeks of age. Relative abundance of renal cortical (**B**) eNOS, (**C**) nNOS, (**D**) PRMT1, (**E**) DDAH1, and (**F**) DDAH2. *n* = 7–8/group; * *p* < 0.05 versus control; # *p* < 0.05 versus HF; † *p* < 0.05 versus BPA; ‡ *p* < 0.05 versus HF + BPA.

**Figure 3:**
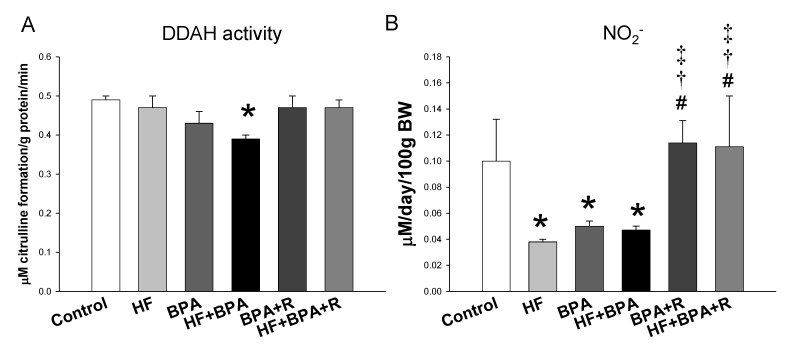
Effects of maternal high-fat diet (HF), bisphenol A (BPA), and resveratrol (R) on (**A**) renal DDAH activity and (**B**) urinary NO_2_^−^ levels in male offspring at 16 weeks of age. *n* = 7–8/group; * *p* < 0.05 versus control; # *p* < 0.05 versus HF; † *p* < 0.05 versus BPA; ‡ *p* < 0.05 versus HF + BPA.

Next, we examined the expression of the proteins involved in the ADMA–NO pathway. As shown in Figure 2, the renal endothelial NO synthase (eNOS) protein level was lower in the HF + BPA treated offspring vs. controls, which was prevented by resveratrol treatment. Similarly, combined HF and BPA exposure caused a reduction of the neuronal NO synthase (nNOS) protein abundance, while this reduction was mitigated by resveratrol treatment. Additionally, our data demonstrated that protein levels of PRMT1 (ADMA-synthesizing enzyme), and DDAH-1 and -2 (ADMA-metabolizing enzymes) were not different among the six groups. Nevertheless, we found renal DDAH activity was lower in the HF + BPA group than that in the controls (Figure 3A). We measured the stable metabolite of the NO and NO_2_^−^ levels in the urine to reflect renal NO bioavailability. The urinary NO_2_^−^ level was lower in the HF, BPA, and HF + BPA groups than that in the controls (Figure 3B). However, resveratrol significantly increased the urinary NO_2_^−^ level in the HF + BPA + R group vs. the HF + BPA group.

### 2.4. Immunohistochemistry Staining of 8-OHdG

**Figure 4:**
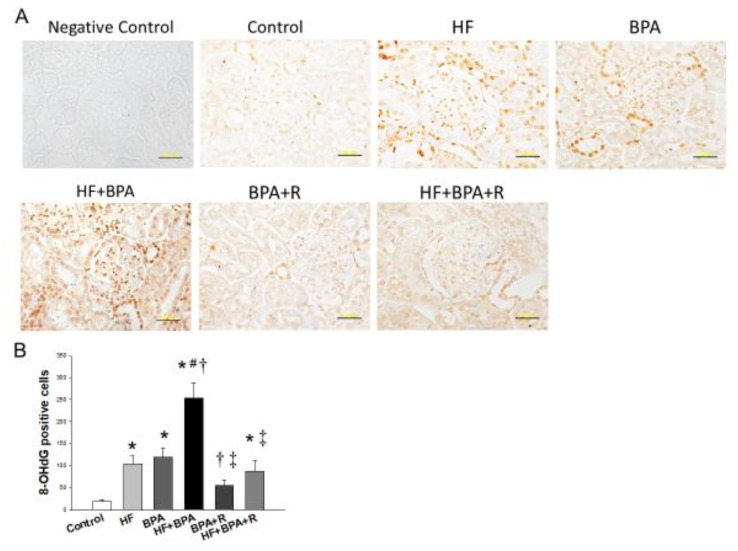
(**A**) Light micrographs illustrating immunostaining for 8-hydroxydeoxyguanosine (8-OHdG) in the kidney in male offspring at 16 weeks of age. Bar = 50 μm. (**B**) Quantitative analysis of 8-OHdG-positive cells per microscopic field (×400). *n* = 5/group; * *p* <0.05 versus control; # *p* <0.05 versus HF; † *p* < 0.05 versus BPA; ‡ *p* < 0.05 versus HF + BPA.

We next investigated oxidative stress damage in offspring kidneys by using immunohistochemistry to analyze 8-hydroxydeoxyguanosine (8-OHdG), an index of oxidative stress-derived DNA damage. As shown in Figure 4A, the immunostaining of 8-OHdG was present in the cytoplasm and nucleus of the glomeruli and renal tubules. There was little staining in the controls (19 ± 4 positive cells), an intermediate level of staining in the HF (104 ± 18 positive cells) and BPA groups (120 ± 21 positive cells), and intense staining in the HF + BPA group (254 ± 35 positive cells) (Figure 4B). Resveratrol treatment significantly reduced 8-OHdG density in the BPA + R group (55 ± 13 positive cells) and the HF + BPA + R group (87 ± 25 positive cells). Our data indicated that combined HF and BPA exposure caused a synergistic effect on oxidative stress damage, which was prevented by resveratrol therapy.

### 2.5. AHR and Its Target Genes

**Figure 5:**
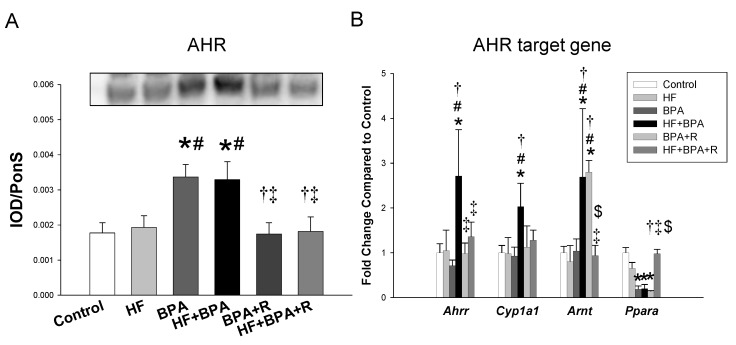
Effects of maternal high-fat diet (HF), bisphenol A (BPA), and resveratrol (R) on (**A**) the aryl hydrocarbon receptor (AHR) protein level and (**B**) mRNA expression of AHR target genes, including *Ahrr, Cyp1a1,* and *Arnt* in male offspring kidneys at 16 weeks of age. *n* = 7–8/group; * *p* < 0.05 versus control; # *p* < 0.05 versus HF; † *p* < 0.05 versus BPA; ‡ *p* < 0.05 versus HF + BPA; $ *p* < 0.05 versus BPA + R.

We further measured the expression of the AHR and AHR target genes. The renal AHR protein level was higher in the BPA and HF + BPA groups compared to that in the controls as well as the HF group (Figure 5A). Additionally, our data show that there was a higher renal mRNA expression of *Ahrr*, *Cyp1a1* and *Arnt* in the HF + BPA-treated offspring compared with those in the controls. We also found that these changes of AHR target genes were restored by resveratrol therapy. 

## 3. Discussion

This study casts a new light on the links of the ADMA–NO pathway, oxidative stress, and the AHR signaling pathway in the kidney by which maternal resveratrol treatment attenuates hypertension programmed by combined BPA and HF exposure in adult male offspring. The key contributions of this work are presented as follows: (1) Maternal BPA exposure exacerbates hypertension programmed by dams exposed to an HF diet in adult male offspring; (2) resveratrol treatment moderates hypertension programmed by BPA or HF + BPA; (3) there was a synergistic effect of the HF diet and BPA exposure on inducing oxidative damage in offspring kidneys, which resveratrol treatment prevented; (4) HF + BPA-induced programmed hypertension is related to decreases of NO bioavailability, increases of oxidative stress, and the activation of the AHR signaling pathway; and (5) resveratrol restores NO bioavailability, reduces oxidative stress, and antagonizes AHR signaling so that HF + BPA-induced hypertension is prevented in adult male offspring.

Early BPA exposure has been associated with an increased risk of developing obesity, diabetes, and cardiometabolic diseases [16,17]. As far as we know, no previous research has investigated whether early exposure to BPA and/or HF diet increases the vulnerability of offspring to hypertension in later life. In the current study, BPA was administered at a dose of 50 μg/kg bodyweight/day based on the estimated human exposure levels and the current oral reference dose set by the US Environmental Protection Agency (USEPA) [18]. Though this dose is likely to be without a considerable risk of deleterious effects during a lifetime [18], our data show mother rats exposed to low doses of BPA during pregnancy and lactation not only caused the elevation of BP in their adult offspring but also aggravated hypertension programmed by the maternal HF diet. Our results are agreement with previous studies showing that different insults could be synergistically contributing to renal programming and programmed hypertension [9,19]. Of note is that the increases of BP were mitigated by maternal resveratrol therapy. Though resveratrol has been reported to lower BP in adult hypertension [20], few studies are available regarding dams exposed to resveratrol protecting programmed hypertension in their adult offspring [12]. To our knowledge, this is the first report showing that resveratrol administration during pregnancy and lactation periods protects adult offspring against hypertension programmed by combined maternal BPA and HF exposure.

Certain mechanisms contributing to the protective effects of resveratrol against HF + BPA-induced hypertension have been observed, such as the reduction of oxidative stress, the restoration of NO bioavailability, and the abrogation of AHR activation. Early-life oxidative stress attributed to NO–ROS imbalance is considered as a key mechanism underlying programmed hypertension [14,21]. A variety of prenatal insults have been associated with oxidative stress to lead to renal programming and programmed hypertension [21], including a maternal HF diet [5]. Though research has reported that BSA exposure can induce oxidative stress, resulting in kidney damage [22], no study to date has examined whether hypertension programmed by maternal BPA exposure is associated with oxidative stress. Our data address that BPA not only increases 8-OHdG staining, an oxidative stress damage marker, but also exacerbates HF-induced increases of 8-OHdG staining. Conversely, resveratrol improves oxidative stress, represented by lower 8-OHdG staining in both the BPA + R and HF + BPA + R groups. These findings tie in well with previous studies wherein the beneficial effects of resveratrol were noted as, at least in part, due to its antioxidant properties [9,10,12,20].

Another protective mechanism of resveratrol on BPA + HF-induced programmed hypertension may be related to the restoration of NO bioavailability. Conflicting a previous study showing that BPA at the dose ranged from 1 to 100 μM increased NO_2_^−^ production in murine endothelial cells [23], we found that the urinary level of NO_2_^−^ was decreased in the BPA-treated offspring. NO is a vasodilator. We observed that both indices of NO bioavailability, l-arginine-to-ADMA ratio and the NO_2_^−^ level, were reduced in the BPA + HF group. Additionally, BPA + HF caused the elevation of BP and was associated with decreased renal protein levels of eNOS and nNOS, an increased plasma ADMA level, and decreased DDAH activity. Our data support a close link between the ADMA–NO pathway and programmed hypertension, which concurs with previous studies from our laboratory and other [14,15]. On the contrary, resveratrol therapy increased the plasma l-arginine level, restored the decreased protein levels of eNOS and nNOS, improved renal DDAH activity, and the increased NO_2_^−^ level. Our data are in agreement with previous studies showing that the early restoration of the ADMA–NO pathway, prior to hypertension in favor of NO, is able to prevent the development of hypertension in different hypertensive models [14,24].

Additionally, the beneficial effects of resveratrol therapy could be due to the antagonization of the AHR signaling pathway. Like other EDCs, BPA is a ligand for the AHR. It has been reported that the AHR agonist ligand 2,3,7,8-tetrachlorodibenzo-*p*-dioxin (TCDD) has induced a high BP, and, in this process, AHR target genes like *Ahrr* and *Cyp1a1* may be involved [8,25]. The AHR repressor (AHRR) is an AHR-regulated gene and a negative regulator of the AHR by competing with the AHR for the binding of the AHR nuclear translocator (ARNT) [8]. Our results demonstrated that BPA significantly increased the protein level of the AHR as well as the mRNA expression of *Ahrr*, *Cyp1a1,* and *Arnt* in the HF + BPA-treated offspring kidneys. These findings indicate that BPA activates the AHR signaling pathway, which appears to be correlated with the elevation of BP. Though the AHR has been reported to protect high-fat diet-induced adverse phenotypes in adult mice [26], this notion is not supported by our data, which showed that a maternal HF diet had no effect on the AHR signaling pathway in the offspring. Nevertheless, these HF + BPA-induced increases of the AHR protein and the mRNA expression of *Ahrr*, *Cyp1a1,* and *Arnt* were restored by resveratrol therapy. The present observations suggest that resveratrol may act as an AHR antagonist and inhibit BPA-induced AHR target gene expression. 

One limitation in the current study is that we did not conduct control + R, BPA + R, and HF + R groups. One reason is because resveratrol has no prior history of toxicity in humans [27]. Additionally, we mainly focused on studying the reprogramming effect of resveratrol on the two-hit HF + BPA model rather than the one-hit model. However, whether maternal resveratrol therapy might cause long-term reprogramming changes in offspring prenatally exposed to BPA or HF alone remains to be clarified. Next, we did not examine different doses and exposures of BPA. Given that BPA effects could be very different for low and high doses [28], it would be interesting to determine whether various exposure protocols of BPA lead to differential phenotypes of adult offspring. Moreover, we did not examine other organs involved in BP control, such as the vasculature, heart, and brain. The beneficial effects of maternal resveratrol therapy might be derived from these organs. 

## 4. Materials and Methods 

### 4.1. Animal Models

This study was approved by the Institutional Animal Care and Use Committee of the Kaohsiung Chang Gung Memorial Hospital (Permit number: 2018061303). The present study followed the Guide for the Care and Use of Laboratory Animals of the National Institutes of Health. Virgin Sprague Dawley (SD) rats (12–16 weeks old) were obtained from BioLASCO Taiwan Co., Ltd. (Taipei, Taiwan) and housed in an Association for Assessment and Accreditation of Laboratory Animal Care International (AAALAC)-accredited animal facility in our hospital with a controlled temperature and light cycle (12/12 light cycle). Male SD rats were kept with individual females until mating was confirmed by the examination of a vaginal plug. In order to equally receive maternal pup care and a quantity of milk, litters were standardized to eight pups per litter at birth. Only male offspring were selected from each litter and used in subsequent experiments, as males are prone to develop hypertension at an earlier age compared to females [29]. 

Male offspring were assigned to six groups (*n* = 8 for each group): Control, HF, BPA, HF + BPA, BPA + R, and HF + BPA + R. Maternal rats received a regular rat chow (Fwusow Taiwan Co., Ltd., Taichung, Taiwan; 52% carbohydrates, 23.5% protein, 4.5% fat, 10% ash, and 8% fiber) or a high-fat diet (HF; D12331, Research Diets, Inc., New Brunswick, NJ, USA; 58% fat (hydrogenated coconut oil) plus high sucrose (25% carbohydrate)) during the entire period of pregnancy and lactation [5]. To construct the BPA exposure model, pregnant rats received an oral administration of BPA (50 μg/kg/day dissolved in corn oil; Aldrich Chemical Co., Milwaukee, WI) or vehicle during pregnancy and lactation. The dose of BPA used here was based on a previous study [30]. In addition to HF and BPA exposure, six mother rats in the R group received resveratrol 50mg/L in drinking water (2.5mg/kg/day) during the pregnancy and lactation periods. At the same dose and therapeutic period, resveratrol has been shown effective for the prevention of hypertension in different developmental programming models [9,31].

Using an indirect tail-cuff method (BP-2000, Visitech Systems, Inc., Apex, NC, USA), we measured BP in conscious rat offspring at 3, 4, 8, 12, and 16 weeks of age, as previously described [5]. Rats were acclimated to restraint and tail-cuff inflation for 1 week prior to the experiment. All rats were sacrificed at 16 weeks of age. Rats were anesthetized using an intraperitoneal injection of ketamine (50 mg/kg) and xylazine (10 mg/kg), and then they were euthanized by an intraperitoneal overdose of pentobarbital. Kidneys and heparinized blood samples were collected at the end of the study.

### 4.2. High-Performance Liquid Chromatography (HPLC)

Using HPLC with the o-phtalaldehyde-3-mercaptoprionic acid derivatization reagent described previously, we measured several elements of the NO pathway, including l-citrulline, l-arginine, ADMA, and SDMA [5]. Standards contained concentrations of 1–100 mM l-citrulline, 1–100 mM l-arginine, 0.5–5 mM ADMA, and 0.5–5 mM SDMA were used. 

### 4.3. Quantitative Real-Time Polymerase Chain Reaction (PCR)

RNA was extracted from the kidney cortex as described previously [5]. Three AHR target genes were analyzed, including *Ahrr*, *Cyp1a1*, and *Arnt*. The 18S rRNA gene (*Rn18s*) was used as an internal control gene. Primer sequences are listed in Table 3. RNA expression levels were normalized to 18S rRNA levels and calculated according to the ΔΔCt method. 

### 4.4. Western Blot

A Western blot analysis was performed using the methods published previously [5]. Briefly, protein (200 μg of kidney cortex) from the supernatant of each sample was separated by SDS-PAGE and transferred onto a nitrocellulose membrane (GE Healthcare Bio-Sciences Corp., Piscataway, NJ, USA). The membranes were incubated with a Ponceau S red (PonS) stain solution (Sigma-Aldrich, St. Louis, MO, USA) for 10 minutes on the rocker. After blocking with phosphate-buffered saline-Tween (PBS-T) containing 5% dry milk, the membranes were incubated with a primary antibody. We used the following primary antibodies: A mouse monoclonal anti-endothelial NOS antibody (1:250 dilution, 1-hour incubation; Transduction Laboratories, Lexington, KY), a mouse monoclonal anti-neuronal NOS antibody (1:200 dilution, overnight incubation; Santa Cruz Biotechnology Inc, Santa Cruz, CA), a rabbit anti-human protein arginine methyltransferase-1 (1:200; Millipore, Billerica, MA), a goat anti-rat dimethylarginine dimethylamonihydrolase-1 (1:500, overnight incubation; Santa Cruz Biotechnology Inc), a goat anti-rat dimethylarginine dimethylamonihydrolase-2 (1:100, overnight incubation; Santa Cruz Biotechnology Inc), and a rabbit anti-rat AHR antibody (1:1000, overnight incubation; NB100-2289, Novus Biologicals, Littleton, CO, USA). Following five washes with 0.1% Tween-Tris-buffered saline (TBS-T), the membranes were incubated for 1 h with a horseradish peroxidase-labeled secondary antibody diluted 1:1000 in TBS-T. Bands were visualized using SuperSignal West Pico reagent (Pierce; Rockford, IL, USA). Band density was calculated as the integrated optical density (IOD) minus the background value. The density of Ponceau S staining was used to correct for variations in total protein loading. The protein abundance was represented as IOD/PonS. 

### 4.5. Immunohistochemistry Staining

Paraffin-embedded tissue was sectioned at a 3 μm thickness. Tissue slides were deparaffinized with xylene and rehydrated in a series of ethanol solutions with decreasing concentrations. Following blocking with immunoblock (BIOTnA Biotech., Kaohsiung, Taiwan), the sections were incubated for 2 h at room temperature with an anti-8-OHdG antibody (clone #N45.1, 1:100, JaICA, Shizuoka, Japan). Immunoreactivity was revealed using the polymer-horseradish peroxidase (HRP) labeling kit (BIOTnA Biotech) and 3,3′-diaminobenzidine (DAB) as the chromogen. An identical staining protocol omitting incubation with a primary antibody was employed to prepare samples that were used as negative controls. A quantitative analysis of positive cells per microscopic field (X400) in the renal sections was performed as we described previously [10].

### 4.6. Statistical Analysis

A statistical analysis was conducted with a one-way analysis of variance (ANOVA) with a Tukey post hoc test for multiple comparisons. BP was analyzed by a two-way repeated-measures ANOVA with a Tukey post hoc test. All values are reported as mean ± SEM. A probability value <0.05 was considered statistically significant. All analyses were performed using the SPSS 14.0 (IBM, Armonk, NY, USA). 

## 5. Conclusions

In conclusion, maternal BPA exposure exacerbates hypertension programmed by HF intake in male adult offspring. Our data highlight that early-life environmental EDC exposure may interact with in utero nutritional insults, leading to adverse outcomes in later life. Resveratrol, an antioxidant and an AHR antagonist, may serve as a reprogramming strategy to prevent hypertension programmed by HF + BPA exposure. Several important mechanisms are involved in the protective actions of resveratrol against hypertension in adult offspring, including reducing oxidative stress, restoring NO bioavailability, and antagonizing the AHR signaling pathway. However, it is not possible to reach therapeutically relevant doses through the daily uptake of conventional foods (e.g., grapes) or beverages (e.g., wine). Therefore, clinical trials with the aim of developing resveratrol-enriched supplements to determine effective interventions for the therapy of specific diseases in pregnancy are urgently needed. With a better understanding of the environment–diet interactions that underlie hypertension programmed by combined BPA and HF exposure, our results can aid in developing effective reprogramming strategies to prevent hypertension and related disorders.

## Figures and Tables

**Table 1:** Morphological values and blood pressures in different experimental groups.

	Control	HF	BPA	HF + BPA	BPA + R	HF + BPA + R
*n*	8	8	8	8	8	8
Mortality	0%	0%	0%	12.5%	0%	0%
Body weight (BW) (g)	553 ± 12	533 ± 21	501 ± 11	558 ± 16	553 ± 11	525 ± 13
Left kidney weight (g)	2.22 ± 0.1	1.96 ± 0.07	2.06 ± 0.09	2.21 ± 0.06	2.26 ± 0.08	2.00 ± 0.06
Left kidney weight/100g BW	0.4 ± 0.02	0.37 ± 0.01	0.41 ± 0.01	0.40 ± 0.02	0.41 ± 0.01	0.38 ± 0.01
Systolic blood pressure (mmHg)	141 ± 1	146 ± 0.4 *	151 ± 1 *#	162 ± 1 *#†	141 ± 1 #†‡	152 ± 1 *#$

*n* = 8/group; * *p* < 0.05 versus control; # *p* < 0.05 versus HF; † *p* < 0.05 versus BPA; ‡ *p* < 0.05 versus HF + BPA; $ *p* < 0.05 versus BPA + R.

**Table 2:** Plasma levels of NO-related elements in different experimental groups.

	Control	HF	BPA	HF + BPA	BPA + R	HF + BPA + R
l-citrulline (μM)	56.4 ± 5.8	45 ± 2	52.9 ± 3.6	42.8 ± 4.5	48.8 ± 8.6	67.3 ± 4.3 ‡
l-arginine (μM)	153.6 ± 12.8	125.7 ± 9.6	137.1 ± 12.9	141.9 ± 7.2	175 ± 17	191.7 ± 11.7 #†
ADMA (μM)	1.66 ± 0.21	1.36 ± 0.1	2.14 ± 0.22	2.67 ± 0.27 *#†	2.26 ± 0.21 #‡	2.8 ± 0.2 *#
SDMA (μM)	0.38 ± 0.07	0.31 ± 0.05	1.01 ± 0.12 *#	0.74 ± 0.16*#	0.58 ± 0.1 †	0.7 ± 0.1 #
l-arginine-to-ADMA ratio (μM/μM)	99.3 ± 10.8	97.2 ± 12.9	66.2 ± 6.6	58.2 ± 9.4*#	79.5 ± 7	69.9 ± 6.6

*n* = 7–8/group; * *p* <0.05 versus control; # *p* < 0.05 versus HF; † *p* < 0.05 versus BPA; ‡ *p* < 0.05 versus HF + BPA.

**Table 3:** Quantitative real-time polymerase chain reaction primers sequences.

Gene	Forward (5′–3′)	Reverse (5′–3′)
*Ahrr*	cagcaacatggcttctttca	tgaagcactgcattccagac
*Cyp1a1*	gcactctggacaaacacctg	atatccaccttctcgcctgg
*Arnt*	gtctccctcccagatgatga	gctggtagccaacagtagcc
*Rn18s*	gccgcggtaattccagctcca	cccgcccgctcccaagatc

*Ahhr* = Aryl hydrocarbon receptor repressor, *Cyp1a1* = Cytochrome P450 CYP 1A1, *Arnt* = Aryl hydrocarbon receptor nuclear translocator, *Ppara* = Peroxisome proliferator-activated receptor α, *Rn18s* = 18S ribosomal RNA (r18S).

## Abbreviations

ADMA	Asymmetric dimethylarginine
AHR	Aryl hydrocarbon receptor
AHRR	Aryl hydrocarbon receptor repressor
ANRT	Aryl hydrocarbon receptor nuclear translocator
BPA	Bisphenol A
DDAH1	Dimethylarginine dimethylamonihydrolase-1
DDAH2	Dimethylarginine dimethylamonihydrolase-2
DOHaD	Developmental origins of health and disease
eNOS	Endothelial NO synthase
EDC	Endocrine disrupting chemical
nNOS	Neuronal NO synthase
NO	Nitric oxide
PRMT1	Protein arginine methyltransferase-1
ROS	Reactive oxygen species
SDMA	Symmetric dimethylarginine
TCDD	2,3,7,8-tetrachlorodibenzo-p-dioxin
8-OHdG	8-Hydroxydeoxyguanosine
